# Antigravity Versus Body-Weight-Supported Treadmill Training in Lower-Limb Arthroplasty Rehabilitation: A Randomized Controlled Pilot Trial

**DOI:** 10.3390/jcm15134918

**Published:** 2026-06-24

**Authors:** Justyna Mazurek, Adam Wrzeciono, Małgorzata Ratajczyk, Olga Witczak, Joanna Szczepańska-Gieracha, Błażej Cieślik

**Affiliations:** 1 Department of Clinical Physiotherapy and Rehabilitation, University Center for Physiotherapy and Rehabilitation, Wroclaw Medical University, 50-368 Wroclaw, Poland; 2 Collegium Medicum, Jan Długosz University in Czestochowa, 42-200 Czestochowa, Poland; 3 Rehabilitation Department, St. Hedwig Hospital, 55-100 Trzebnica, Poland; 4 DSW Ideis University, 53-611 Wroclaw, Poland; 5 Healthcare Innovation Technology Lab, IRCCS San Camillo Hospital, 30126 Venice, Italy

**Keywords:** arthrosis, osteoarthritis, gait training, hip replacement, knee replacement, physiotherapy, lower body positive pressure treadmill training

## Abstract

**Objective:** To evaluate the feasibility of adding antigravity treadmill training (ATT) or harness-based body-weight-supported treadmill training (BWSTT) to standard inpatient rehabilitation after primary hip or knee arthroplasty and to explore preliminary effects on osteoarthritis-related outcomes, balance, and psychological status. **Methods:** In this single-center, assessor-blinded pilot randomized trial, 60 adults within 3 months after primary hip or knee arthroplasty for osteoarthritis were allocated 1:1:1 to ATT, BWSTT, or standard inpatient rehabilitation over 6 weeks. Feasibility outcomes included recruitment, retention, and adherence. ATT and BWSTT additionally included unloading-based treadmill gait training using lower-body positive pressure or a harness system. Exploratory clinical outcomes included WOMAC total and subscale scores, analyzed using baseline-adjusted ANCOVA estimated marginal means. Secondary exploratory outcomes were BBS, FES-I, PHQ-9, and PSS-10. **Results**: Post-intervention data were available for 47 participants, with differential attrition across groups. Exploratory ANCOVA suggested between-group differences for WOMAC total (*p* = 0.004) and WOMAC function (*p* < 0.001). Compared with standard rehabilitation, ATT showed lower adjusted WOMAC total and function scores (both *p* < 0.01). ATT versus BWSTT contrasts for WOMAC total and function were statistically significant in the primary exploratory model but attenuated after hypertension adjustment. Exploratory signals were also observed for BBS and FES-I, although FES-I was less robust in sensitivity analysis. No clear between-group differences were observed for WOMAC pain, stiffness, PHQ-9, or PSS-10. No formal multiplicity adjustment was applied across exploratory endpoints. **Conclusions:** In this single-center pilot randomized trial, ATT suggested preliminary function- and balance-related signals that require confirmation in adequately powered multicenter trials.

## 1. Introduction

Total hip and knee arthroplasty (THA and TKA) are among the most frequently performed and effective surgical procedures for end-stage hip or knee joint degeneration, most commonly osteoarthritis, and procedure volumes continue to rise across health systems internationally [1,2]. These operations generally deliver clinically meaningful improvements in pain, physical function, and health-related quality of life [3]. However, measurable deficits in muscle strength, gait performance, and balance are common and can contribute to functional limitations at discharge and during the first weeks after surgery [4,5,6]. During this period, patients often need to regain safe ambulation, transfers, and basic activities of daily living within a short inpatient window, and inadequate mobility and balance may increase fall risk and delay functional independence [4]. Consequently, structured rehabilitation is widely regarded as a core component of perioperative care to restore mobility and strength, optimize function, and support safe discharge planning [7,8].

Early mobilization and progressive exercise are central elements of arthroplasty rehabilitation [8]. Yet early recovery is frequently constrained by pain, swelling, and neuromuscular impairments, including pronounced quadriceps weakness after TKA [6]. Balance limitations and fear of falling are also clinically relevant and may reduce walking exposure in the first weeks [4]. Unloading-based gait training may address these barriers by enabling treadmill walking with partial reduction in effective body weight. By reducing the mechanical and perceived demands of walking, unloading may enable greater stepping practice and more structured progression when overground gait is limited by symptoms or confidence early after surgery [6,8]. Within contemporary person-centered rehabilitation frameworks, postoperative recovery is conceptualized as an individualized process requiring adaptation of exercise intensity, gait progression, and support strategies to patient-specific functional limitations, tolerance, goals, and comorbidity burden [7,8,9].

Two commonly used unloading modalities are harness-based body-weight-supported treadmill training (BWSTT) and lower-body positive pressure treadmill training (LBPP), also termed antigravity treadmill training (ATT). BWSTT provides adjustable weight support via an overhead suspension system while the patient walks on a motorized treadmill [10]. LBPP treadmills generate graded unloading through a pressurized chamber around the lower body, and biomechanical studies show that ground-reaction forces decrease as support increases [11]. Both approaches allow clinicians to titrate unloading and walking speed in quantifiable steps, which can help structure early gait progression during inpatient rehabilitation and may be particularly relevant in older postoperative populations with variable confidence, balance capacity, and exercise tolerance [10,11,12,13]. However, the modalities differ in how unloading is delivered and how the patient is stabilized: BWSTT uses an overhead harness system, whereas ATT/LBPP provides graded unloading through lower-body positive pressure, which may influence feasibility, tolerance, and perceived safety [10,11,12,13].

Evidence supporting unloading-based treadmill gait training after arthroplasty remains limited and heterogeneous. A randomized pilot study after TKA found antigravity treadmill training feasible and apparently safe, but emphasized the need for larger trials to determine added benefit versus established rehabilitation [12]. A recent scoping review similarly concluded that potential benefits have been reported after knee surgery, but evidence quality is low and superiority over conventional physiotherapy is not established [14]. In related orthopedic populations, ATT studies have reported selected benefits in lower-limb strength, muscle activation, early muscle preservation, or short-term physical function after fracture fixation or arthroplasty, although consistent patient-reported benefits have not been established [15,16,17].

Evidence for BWSTT in postoperative orthopedic rehabilitation is also limited. Randomized evidence exists after THA, although protocols and settings vary [18], and BWSTT has been examined as a feasible inpatient rehabilitation approach after hip fracture, with limited efficacy evidence [13]. In outpatient post-TKA rehabilitation, a large randomized clinical trial found no meaningful short-term differences across several device-based programs, including a body-weight-adjustable treadmill approach [19]. Direct comparisons of BWSTT and ATT within routine inpatient pathways after THA or TKA remain scarce. Because pain, stiffness, and functional limitations remain central patient-reported domains after arthroplasty, the WOMAC was selected as the principal exploratory clinical outcome.

Therefore, this pilot randomized trial aimed to evaluate the feasibility of integrating unloading-based treadmill gait training into a structured inpatient arthroplasty rehabilitation pathway and to generate preliminary estimates of variability and potential treatment effects for future definitive trials. Exploratory objectives were to compare changes in patient-reported osteoarthritis outcomes, including the WOMAC total score and subdomains, as well as balance, concern about falling, and psychological outcomes, between ATT, BWSTT, and standard inpatient rehabilitation.

## 2. Materials and Methods

### 2.1. Study Design and Setting

This was a three-arm, parallel-group randomized controlled pilot trial with blinded outcome assessment, conducted at St. Hedwig of Silesia Hospital in Trzebnica (Poland). We targeted 20 participants per group, consistent with recommendations for pilot RCTs to inform feasibility and variance estimates [20,21]. The pilot sample was intended to provide preliminary estimates of recruitment and retention rates, intervention adherence, and the variability of WOMAC outcomes to support planning and sample size estimation for a future definitive multicenter trial. Participants were randomized 1:1:1 using computer-generated permuted blocks with allocation concealment via sequentially numbered opaque envelopes prepared off-site [22]. Although outcome assessors were blinded to treatment allocation, a formal assessment of blinding integrity was not performed. Assessments were performed at baseline (T0) and after 6 weeks (T1). The protocol was approved by the Research Ethics Committee at Jan Dlugosz University in Czestochowa, Poland (KE-U/74/2025). The trial was prospectively registered (NCT07040878), and reporting follows CONSORT [23].

### 2.2. Participants

Consecutive patients aged 60–85 years, within 3 months after primary total hip or knee arthroplasty, admitted to the study rehabilitation unit were screened for eligibility. Both THA and TKA participants were eligible because the rehabilitation pathway and study interventions were integrated within the same inpatient postoperative rehabilitation program. Inclusion required the ability to provide informed consent and comply with study procedures. Exclusion criteria were cognitive impairment precluding independent questionnaire completion; a history of consciousness disturbances, a psychotic disorder, a bipolar disorder, or other severe psychiatric illness; a functional status preventing independent ambulation (wheelchair-bound or bedridden; the use of crutches or a walker was permitted); and the refusal or withdrawal of consent at any stage. Sixty patients met the criteria and were enrolled; all provided written informed consent.

### 2.3. Interventions

All participants received the same therapist-supervised inpatient rehabilitation for 6 weeks (five sessions/week): a 120 min/day of kinesitherapy, a 30 min/day of ergotherapy targeting activities of daily living, and individualized physical-therapy procedures (e.g., laser, magnetic, or electrotherapy) applied according to clinical need. Selection of adjunct physical-therapy modalities was based on routine clinical assessment by the treating rehabilitation physician and physiotherapist, considering pain severity, postoperative soft-tissue status, and functional limitations. Outside of gait training, concomitant care was identical across groups and delivered by licensed physiotherapists using written protocols with session-by-session adherence logs.

In the ATT arm, walking was performed on a differential-air-pressure device (BTL R-Force, BTL Industries Limited, Stevenage, United Kingdom) that reduced effective body weight while preserving kinematics; treadmill speed was individualized at the first session and increased by 0.5 km/h every two weeks as tolerated, with unloading tapered across the program (around 60% in weeks 1–2, 40% in weeks 3–4, 20% in weeks 5–6) and adjusted for pain, comfort, perceived exertion, gait quality, and safety.

In the body-weight-supported treadmill arm, gait training was performed on a conventional treadmill (Biodex Gait Trainer™ 3, Biodex Medical Systems, Inc., Shirley, NY, USA) with an overhead harness providing partial body-weight support (typically 20–40% initially), with session-to-session adjustments based on pain, comfort, perceived exertion, gait quality, safety, and the ability to maintain safe, symmetrical stepping; speed was individualized at baseline and increased by 0.5 km/h every two weeks as tolerated.

The control group received conventional overground gait practice in hospital corridors under physiotherapist supervision, with assistive devices as required (e.g., crutches or a walker), alongside the same daily kinesitherapy, ergotherapy, and physical-therapy components as the intervention arms.

### 2.4. Outcomes

Feasibility outcomes were predefined to inform the design of a future definitive randomized trial and included the recruitment rate, eligibility rate, retention at 6 weeks, intervention adherence, and reasons for withdrawal. Adherence was defined as the proportion of scheduled intervention sessions completed. Feasibility outcomes were summarized descriptively and were not subjected to formal hypothesis testing.

Exploratory clinical outcomes included the Western Ontario and McMaster Universities Osteoarthritis Index (WOMAC), a 24-item questionnaire covering pain (5 items; 0–20), stiffness (2 items; 0–8), and physical function (17 items; 0–68). Items are rated 0–4 and summed to a total score from 0 to 96, with higher values indicating greater symptom burden and functional limitations [24]. The WOMAC total score was treated as the principal exploratory clinical outcome, whereas WOMAC subscales and secondary outcomes were interpreted as exploratory.

Secondary outcomes included functional balance and psychological status. Functional balance was assessed with the Berg Balance Scale (BBS), a 14-item performance test scored 0–4 per item (total 0–56), sampling static and dynamic tasks such as sit-to-stand, reaching, turning, and single-leg stance; higher scores reflect better balance and lower fall risk, with values below about 45 commonly linked to increased fall risk [25]. Concern about falling was measured with the Falls Efficacy Scale-International (FES-I), a 16-item questionnaire scored 1–4 per item (total 16–64); higher scores indicate greater concern and capture a dimension of fall risk not reflected by performance tests alone [26]. Depressive symptoms were evaluated with the Patient Health Questionnaire-9 (PHQ-9), a 9-item self-report measure yielding a 0–27 total; it indexes the frequency of core DSM depressive symptoms and supports established severity bands [27]. Perceived stress was measured with the 10-item Perceived Stress Scale (PSS-10; total 0–40), which reflects appraisal of stress over the prior month rather than specific events and has good psychometric properties in adult and older populations [28]. All outcomes were administered at baseline and after 6 weeks, using validated Polish versions of the questionnaires.

### 2.5. Data Analysis

Data were analyzed in JASP (version 0.16.4, JASP Team, University of Amsterdam, Amsterdam, The Netherlands). Descriptive baseline comparisons used a one-way ANOVA for continuous variables and *χ*^2^ tests for categorical variables. Analyses followed a modified intention-to-treat (mITT) approach, defined as all randomized participants with a post-baseline (T1) assessment. Session attendance among participants with T1 assessments was 100% and no major protocol deviations occurred, so the per-protocol set was identical to mITT. No imputation was performed; analyses used available cases. The estimand for the exploratory clinical analyses was the between-group difference at post-treatment (T1) adjusting for baseline (T0). Therefore, for each continuous endpoint, we fit an ANCOVA with group as a fixed factor and the corresponding T0 value as a covariate. We report estimated marginal means (EMMs) at T1 with 95% confidence intervals (CIs), overall omnibus tests, and adjusted pairwise mean differences between groups.

Multiplicity for pairwise comparisons within each endpoint was controlled using Tukey’s procedure applied to EMM contrasts. No formal multiplicity adjustment was applied across the multiple exploratory clinical endpoints; therefore, *p* values across endpoints were interpreted descriptively. The two-sided alpha level was 0.05. Effect sizes for omnibus tests are reported as partial eta-squared (*η*p^2^). Model assumptions were checked via the inspection of residuals, the homogeneity of regression slopes, and Levene’s test on model residuals.

As post hoc sensitivity analyses, we examined the robustness of the exploratory clinical findings to the observed baseline imbalance in hypertension and to post-randomization attrition. First, the ANCOVA models were repeated with hypertension status added as an additional binary covariate. Second, the baseline characteristics of participants with and without T1 assessments were compared descriptively overall and within each treatment group using standardized mean differences. These analyses were interpreted descriptively and were used to assess the plausibility and direction of potential bias rather than to redefine the exploratory estimand.

## 3. Results

### 3.1. Participant Flow and Feasibility Outcomes

Of the participants screened, 69% met the eligibility criteria, and 60 participants were randomized 1:1:1 to the ATT, BWSTT, or the control groups (*n* = 20 per arm). In the control group, two participants withdrew after randomization but before the baseline assessment and therefore provided no analyzable study data. Among participants with baseline data who initiated the intervention period, attrition before the post-intervention assessment occurred in 5/20 participants in the ATT group, 3/20 participants in the BWSTT group, and 3/18 participants in the control group. Consequently, post-intervention (T1) outcome data were available for 15 participants (75%) in the ATT group, 17 participants (85%) in the BWSTT group, and 15 participants (83%) in the control group. The available-case exploratory ANCOVA analyses therefore included 47 participants.

**Figure 1:**
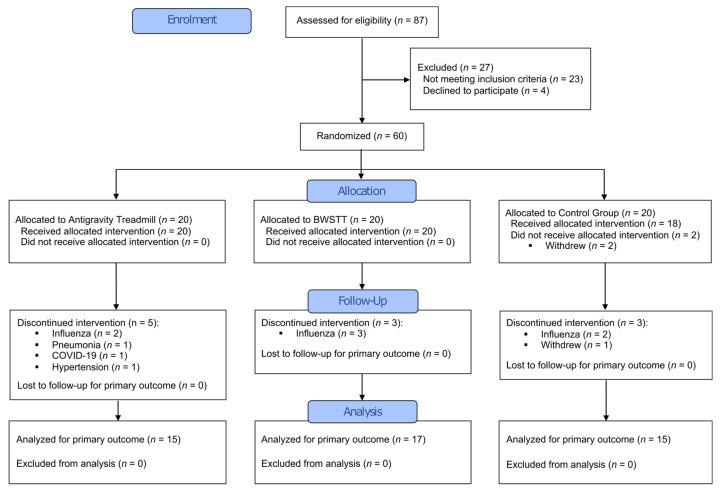
Participant flow diagram. CONSORT flow chart showing screening, randomization (1:1:1 to antigravity treadmill training, body-weight-supported treadmill training, or control), withdrawals, attrition, and numbers included in the modified intention-to-treat analyses.

Among participants who completed the intervention period, adherence to scheduled rehabilitation sessions was high, and no major protocol deviations were identified. Most withdrawals were associated with intercurrent medical illnesses rather than apparent intervention-related adverse events. Retention rates varied across groups and were considered relevant feasibility findings for the planning of a future definitive multicenter trial. Figure 1 shows the CONSORT flow diagram, including the reasons for withdrawal.

### 3.2. Baseline Characteristics

Fifty-eight participants completed the baseline assessment (ATT *n* = 20; BWSTT *n* = 20; control *n* = 18). Groups were comparable in age, body mass, height, BMI (and BMI categories), education, marital status, diabetes, and index joint (knee vs. hip arthroplasty); no between-group differences were detected for these variables (all *p* ≥ 0.48). The only imbalance was for hypertension (ATT 90%, BWSTT 40%, and control 72%; *p* = 0.002). Table 1 presents the detailed baseline data. The baseline characteristics of completers and non-completers are presented in Supplementary Tables S1 and S2.

### 3.3. Functional and Psychological Parameters

At T1, the exploratory baseline-adjusted ANCOVA showed an overall group effect for the principal exploratory clinical outcome, WOMAC total (*F*(2,43) = 6.27, *p* = 0.004, *η*p^2^ = 0.23). On the lower-is-better scale, adjusted mean WOMAC total scores were 9.9 (95% CI 6.5 to 13.3) for ATT, 15.5 (12.3 to 18.6) for BWSTT, and 18.1 (14.8 to 21.5) for the control group. Pairwise contrasts indicated that adjusted WOMAC total scores were lower in the ATT than in the control group (adjusted mean difference −8.2, 95% CI −14.0 to −2.5; *p* < 0.01) and lower in ATT than in BWSTT (−5.6, 95% CI −11.1 to −0.03; *p* = 0.04), whereas BWSTT did not differ from the control group (−2.6, 95% CI −8.2 to 2.9; *p* = 0.49). Full descriptive and inferential results are reported in Table 2.

For the WOMAC subscales at T1, there was also an overall group effect for the WOMAC function (*F*(2,43) = 7.38, *p* < 0.01, *η*p^2^ = 0.26). Adjusted mean scores were 7.7 (95% CI 5.1 to 10.3) for ATT, 12.2 (9.7 to 14.6) for BWSTT, and 14.8 (12.1 to 17.4) for the control group. In the primary exploratory model, ATT showed lower adjusted WOMAC function scores than the control group (−7.1, 95% CI −11.6 to −2.6; *p* < 0.01), BWSTT did not differ from the control group (−2.6, 95% CI −7.0 to 1.7; *p* = 0.33), and ATT showed lower adjusted WOMAC function scores than BWSTT (−4.5, 95% CI −8.8 to −0.1; *p* = 0.04). WOMAC pain and WOMAC stiffness showed no evidence of between-group differences (all adjusted contrasts *p* ≥ 0.17; Table 2).

Among secondary outcomes, ANCOVA indicated an overall group effect for BBS (*F*(2,43) = 4.7, *p* = 0.01, *η*p^2^ = 0.18), with higher adjusted BBS scores in ATT than in the control group (adjusted difference +2.6, 95% CI 0.5 to 4.7; *p* = 0.01). The BWSTT versus control contrast did not reach statistical significance (+1.9, 95% CI −0.2 to 3.9; *p* = 0.07), and ATT versus BWSTT did not differ (+0.68, 95% CI −1.4 to 2.7; *p* = 0.70). FES-I also demonstrated a group effect (*F*(2,43) = 3.7, *p* = 0.03, *η*p^2^ = 0.15), with lower adjusted FES-I scores in ATT than in the control group (−3.1, 95% CI −6.0 to −0.2; *p* = 0.04), with non-significant contrasts for BWSTT versus the control group (−2.5, 95% CI −5.3 to 0.3; *p* = 0.09) and ATT versus BWSTT (−0.6, 95% CI −3.4 to 2.3; *p* = 0.88). PHQ-9 and PSS-10 showed no between-group differences (all adjusted contrasts *p* ≥ 0.78). Detailed estimates and confidence intervals are provided in Table 2.

## 4. Discussion

The present single-center pilot randomized trial examined the feasibility of integrating unloading-based treadmill gait training, delivered as ATT or BWSTT, into inpatient rehabilitation after primary hip or knee arthroplasty. Recruitment and adherence were acceptable, although retention varied across groups. Available-case exploratory analyses indicated lower adjusted WOMAC total and WOMAC function scores in the ATT group than in the standard rehabilitation group, with a similar preliminary pattern for the BBS. These estimates should be interpreted cautiously because the trial was not powered for confirmatory efficacy testing and was affected by a baseline hypertension imbalance, attrition, and multiple exploratory comparisons. In the post hoc sensitivity analyses adjusted for hypertension, the ATT versus control contrasts remained evident for the WOMAC total, WOMAC function, and BBS, whereas the ATT versus BWSTT contrasts for the WOMAC total and WOMAC function were attenuated and no longer statistically significant.

While available evidence remains limited and somewhat inconsistent, our findings are broadly consistent with previous reports suggesting that antigravity treadmill training may support aspects of early postoperative rehabilitation after orthopedic procedures. For example, Kim et al. reported that four weeks of antigravity treadmill training improved selected lower-limb strength and muscle activation outcomes in patients after hip fracture surgery [15]. In a multicenter trial of patients after ankle or tibial plateau fracture fixation, Henkelmann et al. found no between-group differences in patient-reported outcomes at six weeks but reported less thigh muscle atrophy in the antigravity treadmill group, suggesting a potential role in mitigating early disuse-related changes even when short-term self-reported outcomes are similar [16]. In an arthroplasty context, Mikami et al. reported that combining antigravity treadmill training with electrical muscle stimulation helped maintain early postoperative physical function after total hip arthroplasty [17]. However, a recent scoping review concluded that the current evidence base remains heterogeneous and insufficient to establish clear superiority of this modality over conventional rehabilitation [14]. Accordingly, the present results should be interpreted as preliminary data within a still limited evidence base, not as evidence of modality superiority.

From a physiological perspective, the exploratory pattern observed in the ATT group may relate to the combination of graded mechanical unloading and task-specific treadmill walking. By reducing effective body weight and impact-related loading, ATT may allow earlier gait-specific practice under lower mechanical demands, potentially improving patient tolerance and confidence during early postoperative rehabilitation. Evidence from biomechanical laboratory work indicates that antigravity treadmill unloading reduces contact and plantar forces while maintaining broadly recognizable muscle recruitment patterns, although absolute muscle activity may decrease with greater unloading [29]. In arthroplasty populations, adjunctive ATT-based approaches have been reported as feasible and associated with favorable preliminary outcomes in early recovery protocols, including when combined with other modalities such as electrical stimulation, although designs and outcomes vary [17]. However, because gait mechanics, the training dose, perceived exertion, and mechanistic mediators were not quantified, these explanations remain speculative.

The absence of a clear pattern favoring BWSTT over standard rehabilitation should be interpreted cautiously. Evidence on harness-based unloading treadmill approaches in postoperative orthopedic rehabilitation is still sparse and methodologically variable. In a randomized controlled trial after total hip arthroplasty, Hesse et al. reported improvements in hip-specific clinical and gait-related outcomes with treadmill training with partial body-weight support compared with conventional therapy [18]. However, the broader post-arthroplasty exercise literature does not establish a clear optimal device-based approach, and the present pilot sample is too small to support firm conclusions regarding differential modality effects [30]. In related orthopedic populations, BWSTT has also been shown to be feasible during inpatient rehabilitation after a hip fracture, but efficacy data remain limited and require more rigorous trials [13]. Although the observed pattern may reflect differences in how unloading is delivered, this interpretation remains speculative. The hypertension-adjusted sensitivity analysis attenuated the ATT versus BWSTT contrasts for the WOMAC total and WOMAC function; therefore, the present data should not be interpreted as evidence of the superiority of ATT over BWSTT.

The available-case analysis suggested a possible difference in FES-I scores in the ATT group relative to standard rehabilitation, but this contrast was attenuated after adjustment for hypertension; therefore, any inference regarding fall-related confidence should be considered less robust than the WOMAC function and BBS findings. Nevertheless, fall-related confidence remains clinically relevant after arthroplasty because the fear of falling can restrict activity and participation [31]. Antigravity treadmill unloading may provide a lower-load environment with reduced physiological demands, potentially supporting confidence and engagement in gait practice even when pain outcomes are unchanged [32,33]. Broader psychological outcomes (PHQ-9 and PSS-10) did not differ between groups, so any inference regarding mental health effects remains tentative. Future trials should assess whether perceived safety, tolerability, adherence, and patient experience mediate functional gains [34].

### 4.1. Clinical and Feasibility Implications

Clinically, these findings support further investigation of ATT as a feasible adjunct to inpatient arthroplasty rehabilitation, but they do not establish superiority over existing rehabilitation strategies. The integration of unloading-based treadmill interventions appeared practicable, with high adherence among completers and no major protocol deviations; however, retention varied across groups and should inform retention procedures, sample-size inflation, and prespecified missing-data strategies in a future definitive multicenter trial.

The exploratory clinical pattern suggests that unloading-based gait practice may influence perceived function and balance during early recovery, whereas any signal for fall-related confidence appears less robust after adjustment for hypertension. Given that pain and stiffness did not differ between groups, the potential value of ATT may relate more to facilitating earlier lower-load gait practice, confidence, and functional engagement than to direct symptom relief. Within individualized rehabilitation frameworks, implementation would require patient-specific titration of unloading and treadmill speed to balance tolerability with an adequate training stimulus. This approach is consistent with individualized rehabilitation plans and learning rehabilitation system perspectives, which place rehabilitation technologies within individualized, goal-oriented care pathways adapted to patients’ functioning, tolerance, environment, and the service context [9,35]. Greater support generally reduces the physiological demand and plantar forces but may also attenuate lower-limb muscle activity if excessive [29,33]. These implications require confirmation in larger multicenter trials with longer follow-up and stronger control of prognostic covariates and missing data.

### 4.2. Study Limitations

This pilot randomized trial was conducted at a single center with a modest sample size; therefore, the effect estimates are imprecise and may not generalize to other institutions, staffing models, or rehabilitation pathways. The intervention was delivered within an intensive 6-week inpatient rehabilitation pathway, which may not reflect the shorter-stay arthroplasty pathways commonly used in other healthcare systems. Therefore, the feasibility, adherence, and exploratory clinical estimates may not directly generalize to short-stay inpatient, outpatient-based, or home-based rehabilitation models. Follow-up ended at discharge, so the durability of effects and post-discharge outcomes, including falls, were not assessed. The exploratory clinical analyses used available post-treatment cases without outcome imputation. Although most withdrawals were related to intercurrent illnesses rather than apparent intervention intolerance, completers and non-completers differed on selected baseline functional measures, indicating that an attrition-related bias cannot be excluded. A substantial baseline imbalance in hypertension may also have influenced outcomes. Although hypertension-adjusted sensitivity analyses were added, these analyses do not eliminate the risk of residual confounding in a small pilot trial. Randomization was not stratified by index joint (hip vs. knee), which may have introduced additional prognostic heterogeneity. No formal multiplicity adjustment was applied across the multiple exploratory endpoints, so *p* values should be interpreted descriptively. In addition, the modality-specific proportions of adjunct physical therapy procedures were not available in sufficient detail; therefore, unmeasured differences in co-interventions or dosing may have influenced the exploratory between-group estimates. Finally, participants were not blinded, blinding integrity was not formally assessed, and several outcomes were patient-reported, increasing the susceptibility to expectancy, response, and ascertainment biases. The BBS also showed high post-treatment values, suggesting possible ceiling effects.

## 5. Conclusions

In this single-center pilot randomized trial, integrating unloading-based treadmill interventions into an intensive inpatient rehabilitation pathway after hip or knee arthroplasty appeared feasible, although retention varied across groups. Exploratory available-case analyses suggested a potential ATT signal for lower WOMAC total and WOMAC function scores relative to standard rehabilitation, together with a possible balance-related signal on the BBS. For the ATT versus control contrasts, these findings were generally directionally consistent after adjustment for hypertension, whereas the ATT versus BWSTT contrasts were attenuated and no longer statistically significant. The FES-I signal was less robust after the hypertension adjustment, and the WOMAC pain, WOMAC stiffness, PHQ-9, and PSS-10 did not show clear between-group differences. Overall, these clinical findings should be considered preliminary and hypothesis-generating. Larger multicenter trials with longer follow-up, prespecified covariate adjustments, explicit multiplicity strategies, stratification by index joint, and prespecified missing-data procedures are warranted.

## Figures and Tables

**Table 1:** Baseline characteristics of participants.

Variable		ATT	BWSTT	Control Group	*p* Value
*n*		20	20	18	-
Age, years		70.3 (8.9)	72.8 (7.2)	70.4 (4.8)	0.48
Body mass, kg		78.1 (14.7)	78.6 (11.3)	78.2 (12.1)	0.99
Body height, cm		164.4 (9.3)	166.7 (9.1)	164.4 (5.8)	0.60
BMI		28.8 (4.5)	28.3 (3.6)	29.0 (4.9)	0.86
	Normal (BMI 18.5–24.9), *n* (%)	2 (10)	3 (15)	2 (11)	0.79
	Overweight (BMI 25–29.9), *n* (%)	14 (70)	10 (50)	11 (61)	
	Obese (BMI > 30), *n* (%)	4 (20)	7 (35)	5 (28)	
Education, *n* (%)					
	Primary/vocational	8 (40)	8 (40)	7 (39)	0.90
	Secondary	9 (45)	8 (40)	6 (33)	
	Higher	3 (15)	4 (20)	5 (28)	
Marital status, *n* (%)					
	Married	11 (55)	13 (65)	8 (45)	0.63
	Single	2 (10)	3 (15)	4 (22)	
	Widowed	7 (35)	4 (20)	6 (33)	
Hypertension, *n* (%)		18 (90)	8 (40)	13 (72)	0.002
Diabetes mellitus, *n* (%)		6 (30)	6 (30)	4 (22)	0.82
Diagnosis, *n* (%)					
	Knee arthroplasty	10 (50)	9 (45)	8 (44)	0.81
	Hip arthroplasty	10 (50)	11 (55)	10 (56)	

Values are presented as mean (SD) or *n* (%). *p* values are from one-way ANOVA for continuous variables and χ^2^ tests for categorical variables. ATT, antigravity treadmill training; BWSTT, body-weight-supported treadmill training; BMI, body mass index; SD, standard deviation.

**Table 2:** Baseline-adjusted outcomes at discharge by group.

Outcome	Group	Mean (SD)			Post EMM, (95% CI)	Between-Groups Mean Difference (95% CI; *p* Value)		
		Baseline	6 Weeks	Change (%)		BWSTT − Control	ATT − Control	ATT − BWSTT
**WOMAC** **total**	ATT	38.3 (15.5)	9.8 (6.1)	74 ↓	9.9 (6.5, 13.3)		−8.2 (−14.0, −2.5; <0.01)	−5.6 (−11.1, −0.03; 0.04)
	BWSTT	40.6 (18.2)	16.0 (10.4)	61 ↓	15.5 (12.3, 18.6)	−2.6 (−8.2, 2.9; 0.49)		
	Control	35.2 (13.8)	17.7 (8.2)	50 ↓	18.1 (14.8, 21.5)			
**WOMAC function**	ATT	29.5 (12.0)	7.9 (4.6)	73 ↓	7.7 (5.1, 10.3)		−7.1 (−11.6, −2.6; <0.01)	−4.5 (−8.8, −0.1; 0.04)
	BWSTT	30.5 (13.3)	12.4 (8.0)	59 ↓	12.2 (9.7, 14.6)	−2.6 (−7.0, 1.7; 0.33)		
	Control	27.1 (10.1)	14.3 (6.9)	47 ↓	14.8 (12.1, 17.4)			
**WOMAC pain**	ATT	6.2 (4.2)	1.3 (1.7)	79 ↓	1.5 (0.6, 2.4)		−0.7 (−2.3, 0.8; 0.46)	−1.1 (−2.6, 0.4; 0.17)
	BWSTT	7.3 (4.3)	2.8 (2.6)	62 ↓	2.6 (1.7, 3.4)	0.4 (−1.1, 1.9; 0.81)		
	Control	5.8 (4.0)	2.2 (1.8)	62 ↓	2.2 (1.3, 3.1)			
**WOMAC stiffness**	ATT	2.6 (2.0)	0.6 (0.7)	77 ↓	0.7 (0.2, 1.1)		−0.5 (−1.3, 0.2; 0.20)	−0.1 (−0.8, 0.6; 0.91)
	BWSTT	2.7 (2.1)	0.8 (1.0)	70 ↓	0.8 (0.4, 1.2)	−0.4 (−1.1, 0.3; 0.35)		
	Control	2.3 (2.0)	1.2 (1.1)	48 ↓	1.2 (0.8, 1.6)			
**BBS**	ATT	43.6 (8.7)	53.7 (2.6)	23 ↑	54.0 (52.7, 55.2)		2.6 (0.5, 4.7; 0.01)	0.68 (−1.4, 2.7; 0.70)
	BWSTT	45.8 (7.1)	53.4 (3.2)	17 ↑	53.3 (52.1, 54.5)	1.9 (−0.2, 3.9; 0.07)		
	Control	46.6 (5.0)	51.6 (3.1)	11 ↑	51.4 (50.2, 52.7)			
**FES-I**	ATT	35.8 (10.1)	23.6 (4.5)	34 ↓	23.1 (21.4, 24.8)		−3.1 (−6.0, −0.2; 0.04)	−0.6 (−3.4, 2.3; 0.88)
	BWSTT	36.4 (9.9)	23.7 (5.8)	35 ↓	23.7 (22.1, 25.3)	−2.5 (−5.3, 0.3; 0.09)		
	Control	33.9 (8.8)	25.7 (5.6)	24 ↓	26.2 (24.5, 27.9)			
**PHQ-9**	ATT	7.1 (3.9)	3.4 (2.5)	52 ↓	3.5 (2.3, 4.6)		−0.6 (−2.6, 1.5; 0.78)	−0.1 (−2.1, 1.9; 0.99)
	BWSTT	6.3 (4.9)	3.7 (3.3)	41 ↓	3.5 (2.4, 4.7)	−0.5 (−2.5, 1.5; 0.82)		
	Control	5.6 (3.7)	3.9 (3.2)	30 ↓	4.0 (2.8, 5.2)			
**PSS-10**	ATT	21.4 (4.7)	19.7 (3.2)	8 ↓	19.6 (18.1, 21.2)		0.1 (−2.6, 2.7; 1.00)	0.0 (−2.5, 2.5; 1.00)
	BWSTT	21.8 (3.2)	19.7 (3.1)	10 ↓	19.6 (18.2, 21.1)	0.1 (−2.5, 2.7; 1.00)		
	Control	19.8 (5.0)	19.4 (2.5)	2 ↓	19.5 (18.0, 21.1)			

ATT, antigravity treadmill training; BWSTT, body-weight-supported treadmill training; WOMAC, Western Ontario and McMaster Universities Osteoarthritis Index; BBS, Berg Balance Scale; FES-I, Falls Efficacy Scale–International; PHQ-9, Patient Health Questionnaire-9; PSS-10, Perceived Stress Scale; SD, standard deviation; EMM, estimated marginal mean; CI, confidence interval. ↑, increase from baseline; ↓, decrease from baseline. Post hoc sensitivity analyses are presented in Supplementary Tables S1–S3. Baseline comparisons between completers and non-completers indicated that participants without T1 assessments had less favorable baseline profiles for selected functional outcomes, particularly BBS and WOMAC total/function, suggesting that attrition-related bias cannot be excluded. In hypertension-adjusted ANCOVA models, the overall group effect remained for WOMAC total, WOMAC function, BBS, and FES-I. The ATT versus control contrasts remained evident for WOMAC total, WOMAC function, and BBS, whereas the ATT versus BWSTT contrasts for WOMAC total and WOMAC function were attenuated and no longer statistically significant after Tukey adjustment.

## Data Availability

Data are available from the corresponding author upon reasonable request.

## Supplementary Materials

The following supporting information can be downloaded at: https://www.mdpi.com/article/10.3390/jcm15134918/s1, Table S1. Baseline characteristics of completers and non-completers in the overall sample; Table S2. Baseline characteristics of completers and non-completers within each treatment group; Table S3. Post hoc sensitivity ANCOVA additionally adjusted for hypertension.
