# Supplementary material for: Antigravity Versus Body-Weight-Supported Treadmill Training in Lower-Limb Arthroplasty Rehabilitation: A Randomized Controlled Pilot Trial
Source: J Clin Med. 2026 Jun 24;15(13):4918. doi: 10.3390/jcm15134918 (PMC13361619; doi:10.3390/jcm15134918)
Supplement: Supplementary file 1 [file jcm-15-04918-s001.zip › jcm-4317915-SI.pdf]

Supplementary File S1. Attrition and post hoc sensitivity analyses

Supplementary Table S1. Baseline characteristics of completers and non-completers in the overall sample.

**Variable**	**Completers (*n* = 47)**	**Non-completers (*n* = 11)**	**SMD**
Age, years	71.6 (6.7)	69.1 (9.3)	0.353
Female sex	33/47 (70.2%)	7/11 (63.6%)	0.14
BMI, kg/m²	28.5 (4.3)	29.4 (4.2)	-0.208
Knee arthroplasty	21/47 (44.7%)	6/11 (54.5%)	-0.20
Hypertension	33/47 (70.2%)	6/11 (54.5%)	0.328
Diabetes mellitus	13/47 (27.7%)	3/11 (27.3%)	0.009
WOMAC total T0	36.9 (15.1)	43.4 (18.6)	-0.409
WOMAC function T0	28.2 (11.2)	32.9 (14.1)	-0.399
WOMAC pain T0	6.2 (4.1)	7.7 (4.5)	-0.377
WOMAC stiffness T0	2.5 (2.0)	2.7 (2.1)	-0.107
BBS T0	46.2 (6.4)	41.1 (8.7)	0.747
FES-I T0	35.2 (9.4)	36.2 (10.7)	-0.099
PHQ-9 T0	6.1 (4.1)	7.3 (4.5)	-0.278
PSS-10 T0	20.9 (4.6)	21.6 (3.0)	-0.179

Values are presented as mean (SD) for continuous variables or *n* (%) for categorical variables. Completers were defined as participants with available post-treatment assessment at T1. Non-completers were defined as participants with baseline data but missing T1 assessment. Standardized mean differences (SMDs) are presented descriptively to assess baseline differences between completers and non-completers; positive values indicate higher values or proportions among completers, whereas negative values indicate higher values or proportions among non-completers. ATT, antigravity treadmill training; BWSTT, body-weight-supported treadmill training; BMI, body mass index; WOMAC, Western Ontario and McMaster Universities Osteoarthritis Index; BBS, Berg Balance Scale; FES-I, Falls Efficacy Scale–International; PHQ-9, Patient Health Questionnaire-9; PSS-10, Perceived Stress Scale; T0, baseline assessment.

Supplementary Table S2. Baseline characteristics of completers and non-completers within each treatment group.

**Variable**	**ATT**		**BWSTT**		**Control**	
	Completers (*n* = 15)	Non-completers (*n* = 5)	Completers (*n* = 17)	Non-completers (*n* = 3)	Completers (*n* = 15)	Non-completers (*n* = 3)
Age, years	72.2 (8.1)	64.4 (9.6)	71.9 (7.1)	77.3 (7.1)	70.7 (4.7)	68.7 (6.5)
Female sex	11/15 (73.3%)	3/5 (60.0%)	11/17 (64.7%)	1/3 (33.3%)	11/15 (73.3%)	3/3 (100.0%)
BMI, kg/m²	28.4 (4.1)	30.2 (5.8)	28.2 (3.9)	28.9 (1.2)	29.1 (5.2)	28.6 (4.1)
Knee arthroplasty	6/15 (40.0%)	4/5 (80.0%)	9/17 (52.9%)	0/3 (0.0%)	6/15 (40.0%)	2/3 (66.7%)
Hypertension	15/15 (100.0%)	3/5 (60.0%)	7/17 (41.2%)	1/3 (33.3%)	11/15 (73.3%)	2/3 (66.7%)
Diabetes mellitus	6/15 (40.0%)	0/5 (0.0%)	4/17 (23.5%)	2/3 (66.7%)	3/15 (20.0%)	1/3 (33.3%)
WOMAC total T0	36.6 (13.1)	43.4 (22.3)	38.3 (18.4)	53.7 (11.2)	35.6 (13.7)	33.0 (17.1)

WOMAC function T0	28.8 (10.1)	31.6 (17.7)	28.8 (13.3)	40.3 (10.0)	26.9 (10.1)	27.7 (11.9)
WOMAC pain T0	5.5 (4.2)	8.2 (4.0)	6.8 (4.3)	10.7 (2.1)	6.1 (3.8)	4.0 (5.3)
WOMAC stiffness T0	2.3 (2.1)	3.6 (1.5)	2.7 (2.1)	2.7 (2.5)	2.5 (1.9)	1.3 (2.3)
BBS T0	45.4 (7.5)	38.0 (10.3)	46.4 (7.0)	42.3 (7.6)	46.9 (4.5)	45.0 (7.8)
FES-I T0	36.3 (9.8)	34.2 (12.0)	35.3 (10.0)	42.3 (8.3)	34.1 (8.6)	33.3 (11.5)
PHQ-9 T0	6.0 (3.5)	10.2 (3.7)	6.4 (5.0)	6.0 (5.3)	5.9 (3.9)	3.7 (1.5)
PSS-10 T0	21.3 (5.0)	21.4 (4.3)	21.8 (3.5)	21.7 (1.2)	19.3 (5.3)	22.0 (2.6)

Values are presented as mean (SD) for continuous variables or n/N (%) for categorical variables. Completers were defined as participants with available post-treatment assessment at T1. Non-completers were defined as participants with baseline data but missing T1 assessment. These within-group comparisons were interpreted descriptively because of the small number of non-completers in each arm. ATT, antigravity treadmill training; BWSTT, body-weight-supported treadmill training; BMI, body mass index; WOMAC, Western Ontario and McMaster Universities Osteoarthritis Index; BBS, Berg Balance Scale; FES-I, Falls Efficacy Scale–International; PHQ-9, Patient Health Questionnaire-9; PSS-10, Perceived Stress Scale; T0, baseline assessment.

Supplementary Table S3. Post hoc sensitivity ANCOVA additionally adjusted for hypertension.

**Outcome**	**Omnibus group effect**	**Omnibus *p***	**ηp^2**	**Contrast**	**Adjusted mean difference**	**95% CI**	**Tukey-adjusted *p***
WOMAC total	F(2,42)=5.49	0.008	0.207	BWSTT – Control	−2.71	−8.67 to 3.25	0.517
WOMAC total	F(2,42)=5.49	0.008	0.207	ATT – Control	−8.18	−14.19 to −2.17	0.005
WOMAC total	F(2,42)=5.49	0.008	0.207	ATT – BWSTT	−5.47	−12.11 to 1.16	0.124
WOMAC function	F(2,42)=6.41	0.004	0.234	BWSTT – Control	−2.72	−7.40 to 1.95	0.342
WOMAC function	F(2,42)=6.41	0.004	0.234	ATT – Control	−6.96	−11.68 to −2.24	0.002
WOMAC function	F(2,42)=6.41	0.004	0.234	ATT – BWSTT	−4.24	−9.44 to 0.96	0.130
WOMAC pain	F(2,42)=0.98	0.383	0.045	BWSTT – Control	0.30	−1.29 to 1.89	0.889
WOMAC pain	F(2,42)=0.98	0.383	0.045	ATT – Control	−0.69	−2.30 to 0.91	0.551
WOMAC pain	F(2,42)=0.98	0.383	0.045	ATT – BWSTT	−1.00	−2.78 to 0.78	0.371
WOMAC stiffness	F(2,42)=2.15	0.129	0.093	BWSTT – Control	−0.28	−1.03 to 0.47	0.639
WOMAC stiffness	F(2,42)=2.15	0.129	0.093	ATT – Control	−0.65	−1.41 to 0.11	0.108
WOMAC stiffness	F(2,42)=2.15	0.129	0.093	ATT – BWSTT	−0.37	−1.21 to 0.47	0.541
BBS	F(2,42)=5.32	0.009	0.202	BWSTT – Control	1.49	−0.65 to 3.63	0.222
BBS	F(2,42)=5.32	0.009	0.202	ATT – Control	2.87	0.71 to 5.03	0.007

BBS	F(2,42)=5.32	0.009	0.202	ATT – BWSTT	1.38	−1.00 to 3.77	0.345
FES-I	F(2,42)=3.64	0.035	0.148	BWSTT – Control	−2.55	−5.58 to 0.48	0.114
FES-I	F(2,42)=3.64	0.035	0.148	ATT – Control	−3.05	−6.12 to 0.02	0.052
FES-I	F(2,42)=3.64	0.035	0.148	ATT – BWSTT	−0.50	−3.88 to 2.87	0.930
PHQ-9	F(2,42)=0.46	0.632	0.022	BWSTT – Control	−0.82	−2.90 to 1.25	0.604
PHQ-9	F(2,42)=0.46	0.632	0.022	ATT – Control	−0.29	−2.38 to 1.81	0.941
PHQ-9	F(2,42)=0.46	0.632	0.022	ATT – BWSTT	0.53	−1.78 to 2.85	0.841
PSS-10	F(2,42)=0.31	0.733	0.015	BWSTT – Control	−0.51	−3.27 to 2.24	0.894
PSS-10	F(2,42)=0.31	0.733	0.015	ATT – Control	0.46	−2.23 to 3.16	0.909
PSS-10	F(2,42)=0.31	0.733	0.015	ATT – BWSTT	0.97	−2.01 to 3.96	0.710

For each endpoint, the post-treatment value was modelled as the dependent variable, with treatment group as a fixed factor, the corresponding baseline outcome value as a covariate, and hypertension status as an additional binary covariate. Pairwise contrasts are based on estimated marginal means and were adjusted using Tukey's procedure within each endpoint. No formal multiplicity adjustment was applied across the multiple exploratory endpoints; therefore, p values should be interpreted descriptively. ATT, antigravity treadmill training; BWSTT, body-weight-supported treadmill training; WOMAC, Western Ontario and McMaster Universities Osteoarthritis Index; BBS, Berg Balance Scale; FES-I, Falls Efficacy Scale–International; PHQ-9, Patient Health Questionnaire-9; PSS-10, Perceived Stress Scale; CI, confidence interval; ηp^2, partial eta-squared.
